# Satellite imagery pre-processing and feature extraction for the mapping of coastal ecosystems using Google Earth Engine: A workflow for practitioners

**DOI:** 10.1016/j.mex.2025.103516

**Published:** 2025-07-16

**Authors:** Ahmad Badruzzaman, Prawesti Wulandari, Sainal Sainal, Matthew Ashley, Susan Jobling, Melanie C. Austen, Radisti A. Praptiwi

**Affiliations:** a Sustainability Research Cluster and Department of Biotechnology, Universitas Esa Unggul, Jl. Arjuna Utara No. 9, Kebon Jeruk, Jakarta 11510, Indonesia; b Yayasan Puspa Hanuman Indonesia, Bogor, Indonesia; c School of Biological and Marine Sciences, University of Plymouth, Drake Circus, Plymouth, Devon PL4 8AA, United Kingdom; d Civil and Environmental Engineering, College of Design, Engineering and Physical Sciences, and Centre for Pollution Research and Policy, Brunel University London, Uxbridge UB8 3PH, United Kingdom; e PISCES Partnership, Brunel University London, Uxbridge UB8 3PH, United Kingdom; f Research Center for Ecology and Ethnobiology, National Research and Innovation Agency (BRIN), Jl. Raya Jakarta-Bogor KM 46, Cibinong, Bogor 16911, Indonesia

**Keywords:** Earth observation, Remote sensing, Python, GIS, Google Earth Engine, Practitioners, Stakeholders

## Abstract

The use of Google Earth Engine (GEE) is increasingly common in geospatial analysis of satellite images for various environmental management purposes due to its easy accessibility and capabilities to support complex pre-processing and mining of geographic data. In the context of coastal management, GEE provides opportunities for cost-efficient mapping of coastal habitats and their ecosystem service potentials. Understanding the extent of coastal habitats and the spatial and temporal variabilities of their ecosystem services can be useful for management and intervention purposes. GEE is well-suited for this due to its user-friendliness, particularly for non-experts of programming languages, such as area managers and other practitioners. However, there is no specific methodological guideline for the pre-processing and feature extraction of satellite images in GEE that can be readily adopted by these practitioners. This study develops general methodological steps to perform those processes that can be adapted to different management needs. Highlights of this study:

• Steps detailed in this method paper will produce processed satellite images readily applicable for machine learning to classify coastal ecosystems.
• The development of this adaptable workflow can benefit and empower local area managers, particularly in low-resource settings, to conduct monitoring of their area.

## Graphical abstract

**Figure:**
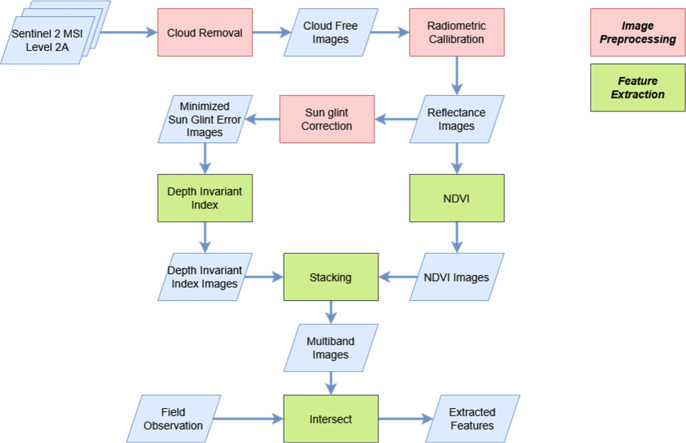

## Specifications table

**Table:** 

**Subject area**	Earth and Planetary Sciences
**More specific subject area**	GIS remote sensing, environmental monitoring.
**Name of your method**	Sentinel-2 Satellite Image Pre-processing and Feature Extraction for Coastal Habitat Mapping.
**Name and reference of original method**	J. W. Rouse, R. H. Haas, J. A. Schell and D. W. Deering. (1974) Monitoring vegetation systems in the great plains with ERTS (Earth Resources Technology Satellite). D. Lyzenga. (1978) Passive remote sensing techniques for mapping water depth and bottom features. https://doi.org/10.1364/AO.17.000379. S. McFeeters. (1996) The use of the Normalized Difference Water Index (NDWI) in the delineation of open water features. https://doi.org/10.1080/01431169608948714. D. R. Lyzenga, N. P. Malinas and F. J. Tanis. (2006) Multispectral bathymetry using a simple physically based algorithm. https://doi.org/10.1109/TGRS.2006.872909. S. Kay, J. Hedley and S. Lavender. (2009) Sun glint correction of high and low spatial resolution images of aquatic scenes: A review of methods for visible and near-infrared wavelengths. https://doi.org/10.3390/rs1040697. P. Wicaksono, P. Aryaguna and W. Lazuardi. (2019) Benthic habitat mapping model and cross validation using machine-learning classification algorithms. https://doi.org/10.3390/rs11111279. R. Martínez Prentice, M. Villoslada Peciña, R. Ward, T. Bergamo, C. Joyce and K. Sepp. (2021) Machine learning classification and accuracy assessment from high-resolution images of coastal wetlands. https://doi.org/10.3390/rs13183669. P. Wicaksono, S. Wulandari, W. Lazuardi and M. Munir. (2021) Sentinel-2 images deliver possibilities for accurate and consistent multi-temporal benthic habitat maps in optically shallow water. https://doi.org/10.1016/j.rsase.2021.100572.
Resource availability	Google Earth Engine; https://scihub.copernicus.eu/

## Background

Coastal ecosystems are essential for the flourishing of human individuals, communities, and societies through their supply of ecosystem services, such as food provisioning, water flow regulation, shoreline protection, and recreational activities [1,2]. The types of ecosystem services and their quantities are particular to specific types of habitats, such as coral reefs and seagrass meadows, due to their inherent ecological structures and functioning [1]. Analysis of ecosystem services can thus be performed at the level of a particular habitat as a service providing unit (SPU) [3]. Therefore, monitoring of coastal ecosystems is important to understand the spatio-temporal dynamics of the ecosystem services they provide and to provide input for appropriate interventions and management of coastal areas [4,5].

In the past decade, remote sensing has increasingly been applied to map coastal ecosystems for monitoring and management purposes [6]. This growing adoption of remote sensing is spurred by its lower resource requirements compared to in-situ monitoring in coastal environment [6,5]. Despite its potential, the uptake of remote sensing applications in the context of low-resource settings still presents several challenges, mainly due to the advanced skills requirement, affordability, and the complex processes of pre-processing and mining of geographic data [5,7]. The use of Google Earth Engine (GEE) can address such challenges, owing to its capabilities in supporting various types of geospatial data and providing a suite of Earth observation data at no-cost [7]. Launched in 2010, GEE is currently the most popular cloud computing platform that provides ease of access for non-experts of programming languages and is capable of operating with common programming languages such as JavaScript and Python [8,9].

However, there is currently no specific methodological guideline on applying image preprocessing and feature extraction steps in GEE to satellite imagery of coastal environments to enable ecosystem mapping, particularly for beginner users. This condition hinders the utilization of the open-access GEE in non-academic low-resource settings, despite the potential of the platform to broaden access to remote sensing technologies and techniques beyond academia [7]. As such, this paper aims to address this knowledge gap, particularly in relation to the needs of practitioners, such as area managers, conservationists, and ecotourism operators, who often lack advanced research methodology training. Enhancing the capacity of local practitioners to address area-specific management challenges is particularly important due to the increasing decision-making responsibilities that these actors have in many coastal and marine contexts [10]. Thus, we specifically provide general methodological steps to perform satellite image pre-processing and feature extraction directly in GEE that can be adapted in various applied contexts for further spatial analysis as required by particular stakeholder needs. The methods described here are designed to enable immediate applications by practitioners in low-resource settings, especially in the Global South. However, the application of our methodological steps still requires a basic knowledge of the Python programming language. There is a variety of resources to learn the language, including from the official Python website (https://www.python.org/about/gettingstarted/) and a range of free online courses [[11], [12], [13]]. GEE itself can also be used in Java programming language, with an online guide for introductory users of GEE, for either Python or Java, available on the GEE website (https://developers.google.com/earth-engine/guides). As indicated in the GEE guide website, the use of Python is more appropriate for more demanding geospatial tasks, such as ecosystem classification based on large datasets, due to the larger libraries for data processing and analysis of satellite images.

## Method details

### Datasets acquisition

#### Remote sensing images

**Fig. 1:**
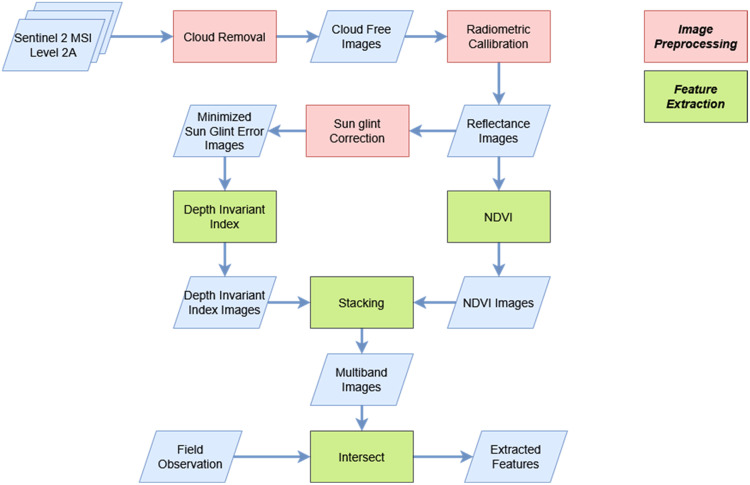
Methodological steps described in this paper.

**Fig. 2:**
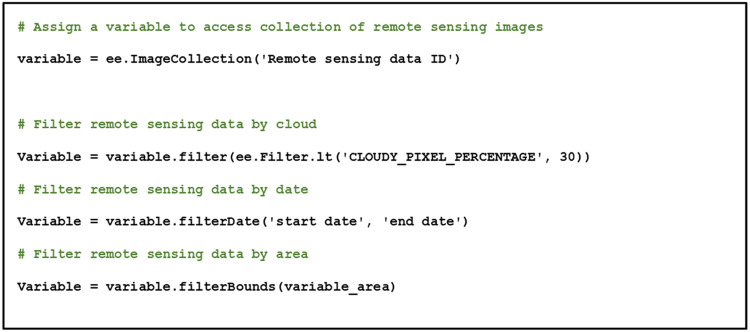
Pseudocode for accessing satellite image collection on Google Earth Engine.

Although various satellite imagery sources can be used in GEE, in this paper we utilized the publicly available Sentinel 2 Multispectral Instrument (MSI) images for the purpose of illustrating the utility of our methodological approach for practitioners (Fig. 1). The Sentinel 2 satellite images should be acquired for the period of interest, according to the specified geographical area in the following website: https://scihub.copernicus.eu/. Instructions to import the obtained satellite images to GEE are available from the following online guides: https://developers.google.com/earth-engine/guides/image_upload. The images should be filtered by cloud coverage under 30 % of the scene and bounded by the study area. If the atmospheric information was unavailable, the usage of Sentinel 2 MSI images should be specified to level 2A data. The atmospheric and terrain can be corrected using the Sen2Cor algorithm with global scale data (low resolution) provided by the European Space Agency (ESA). Although the collected satellite images can be enormous, the usage of Sentinel 2 MSI level 2 data on GEE can simplify the complexity, since Sentinel 2 MSI level 2 periodically records data enabling the acquisition of spectral value under cloud cover from different observation periods with atmospheric and terrain pre-corrections. In GEE, the collection of remote sensing images can be accessed using the function of “ee.ImageCollection”, with the codes shown in Fig. 2.

#### Field observation

**Fig. 3:**
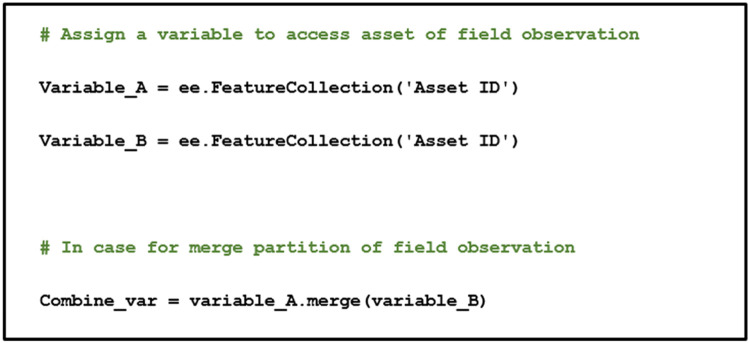
Pseudocode for accessing field observation as feature collection on GEE.

If field observation data, such as coordinates of habitats, is needed for further analysis, this should be based on the commonly used geometrical observation, i.e. point, polyline, and polygon. In general, geotagging must be conducted on the node of every geometrical observation while considering the geospatial resolution of satellite images. Specific to the point observation, the observational classes should be determined by the majority of objects within the geometric resolution of the satellite images. The field observation data uploaded to GEE can be identified as features, using the function of “ee.FeatureCollection” to access the field observation data, with the codes shown in Fig. 3. If the field observation data have nodes exceeding the GEE limit, separate Comma Separated Values (CSV) files for each observational class should be prepared before uploading to the GEE asset (for instance in Excel this can be done by using the concatenate function), which can then be accessed using the codes in Fig. 3.

### Image preprocessing

**Fig. 4:**
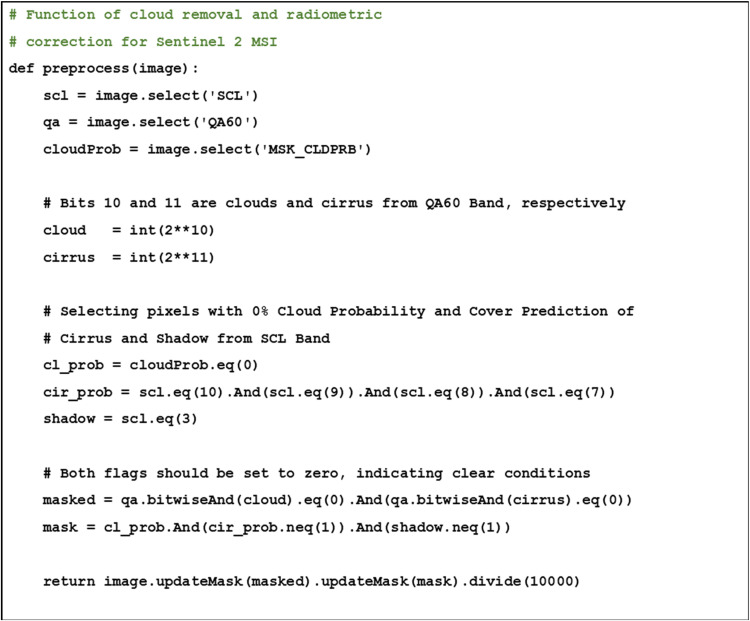
Pseudocode for cloud removal and radiometric correction as proposed scheme on GEE.

**Fig. 5:**
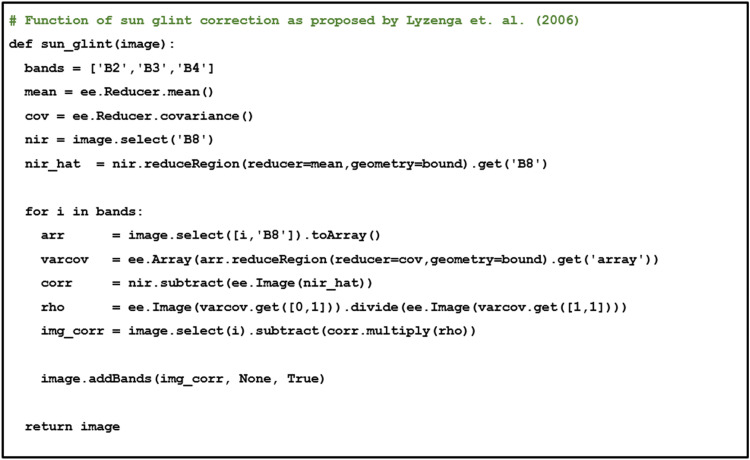
Sun glint correction.

Direct use of raw satellite images for habitat mapping is not recommended due to distortions or noise that may be present in the raw images as a result of geometric distortions [14]. As noise and error are inevitable in any remote sensing data, image preprocessing must be performed to account for them. Fig. 4, Fig. 5 detail the codes to conduct image preprocessing in GEE, further specified in the following separate subsections detailing the rationale behind the GEE codes. The selection of methods arranged in this workflow follows the well-established and commonly used approaches for application in benthic habitat mapping.

#### Cloud removal

Optical remote sensing images are rarely free of clouds, despite the pre-application of the filtering function. Cloud cover and associated shadows could obscure the surface features, hence impacting the analysis accuracy. In Sentinel 2 MSI imagery, cloud and cirrus are recorded in the QA60 Band. Therefore, the cloud mask should be computed by using this band to remove the cloud and cirrus. An alternative approach can be performed by using the MSK_CLDPRB band to select pixels with 0 % probability on both cloud and cirrus. Depending on the analytical needs, combining these approaches can be complementary.

The collected satellite images themselves can compensate for cloud coverage through their combined analysis. However, several regions of the images may overlap and become redundant due to the different satellite observation times. Therefore, statistical computation is required to reduce image collection requirements and enable a single observational image. For this purpose, combining satellite images of the same region should be performed by assuming that the observed reflectance data might be skewed due to abnormal natural phenomena, which act as outliers, hence median values should be used (outlier). Furthermore, this image reduction can be performed either after pre-processing or simultaneously with radiometric correction (described below) to minimize error in this phase, and with codes to do so in GEE displayed in Fig. 4.

#### Radiometric correction

The pixel values of remote sensing images are available in bit format, as Sentinel 2 MSI imagery uses 12-bit values representing surface reflectance, despite GEE relying on the reflectance format. Thus, radiometric correction should be used to convert the pixel values from the bit format to the reflectance format. Let *ρ_i_* as pixel value of *i^th^* band on bit format and *ρ_i_'* as radiometric corrected pixel value of *i^th^* band. Mathematically, radiometric correction for Sentinel 2 MSI imagery can be notated as follows, with the codes to do so in GEE displayed in Fig. 4:

$$\rho_{i}^{\prime }=\rho_{i}÷10000$$

#### Sun glint correction

Coastal environment usually has a wide surface area of water that remote sensing observation can be significantly influenced by sunlight reflection. Therefore, sun glint correction is necessary to correct the specular reflection, hence improving image interpretability and preserving spectral information. Sun glint correction can be performed by using a common sun glint correction method [15,16] that corrects the true color band (RGB) using NIR band. Let $L_{i}^{\prime }$as sun glint corrected of the *i^th^* band, *Li* as pixel value of the *i^th^* band, $L_{NIR}$ as pixel value of NIR band, and ${\bar{L}}_{NIR}$ as the mean value of the observed NIR pixel value on the scene. Mathematically, sun glint correction can be notated as follows, with the GEE codes displayed in Fig. 5.

$$r_{ij}=\frac{Cov\left(i,NIR\right)}{Var\left(NIR,NIR\right)}$$

$$L_{i}^{\prime }=L_{i}-r_{ij}\left[L_{NIR}-{\bar{L}}_{NIR}\right]$$

### Feature extraction

The observed features in coastal areas of interest are derived from satellite images. These features are calculated using band math calculation. The following subsections describe the process of doing so, by selecting features that can be used for further analysis, such as the classification of coastal ecosystems based on machine learning [17,18].

#### Depth invariant bottom index

The reflectance value of coastal features recorded by sensors may be altered due to the effect of the water column. This can induce spectral inconsistencies which may result in misclassification of coastal ecosystems. Depth Invariant Index (DII) can be used to avoid this problem (DII). The method to obtain DII normalizes the effect of water column energy attenuation with the ratio algorithm [19,20]]. Let $\rho_{r}$ as the reflectance of the red band, $\rho_{g}$ as the reflectance of the green band, and $\rho_{b}$ as reflectance of the blue band. Mathematically, DII can be notated as follows:

$$DII_{12}=\frac{\rho_{b}}{\rho_{g}}$$

$$DII_{13}=\frac{\rho_{b}}{\rho_{r}}$$

$$DII_{23}=\frac{\rho_{g}}{\rho_{r}}$$

**Fig. 6:**
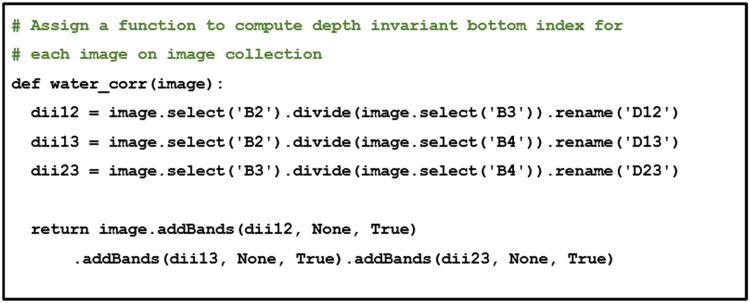
Depth invariant bottom index.

In GEE, DII features are computed using the function of “ee.Image.divide” as shown in Fig. 6.

#### Normalized difference vegetation index (NDVI)

Observational classes of coastal ecosystems often contain chlorophyll from planktonic communities and vegetation, such as mangroves and seagrass [[21], [22], [23]]. Thus, NDVI should be used to quantify the presence of chlorophyll using satellite images [24]. NDVI ranges between −1 to 1, with values below zero indicating either cloud or water, and values near zero indicating bare soil or sand. Mathematically, NDVI is notated as below:

$$NDVI=\frac{\rho_{r}-\rho_{NIR}}{\rho_{r}+\rho_{NIR}}$$

**Fig. 7:**
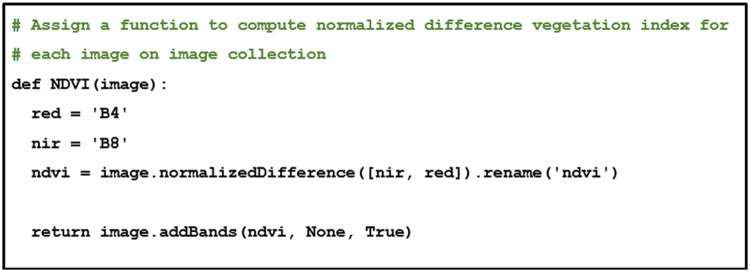
Normalized difference vegetation index.

As with the previous subsection, this feature is computed in a function to calculate each image in the image collection. Specifically, GEE has the function of “ee.Image.normalizedDifference” to simplify the code required to calculate NDVI, as shown in Fig. 7.

#### Normalized difference water index (NDWI)

**Fig. 8:**
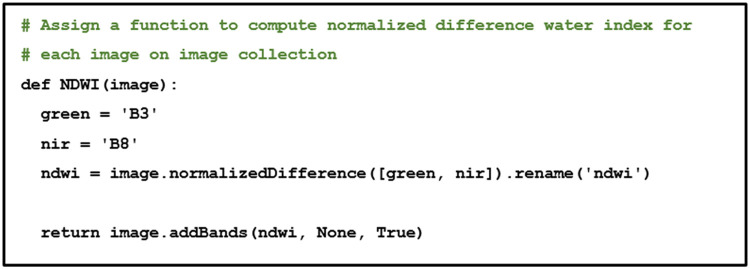
Normalized difference water index.

Specific coastal ecosystems, such as seagrass meadows, intertidal sand and subtidal sediment are often observed beneath the water surface by a satellite. Therefore, NDWI should be performed to ensure observation consistency. NDWI is used to highlight water features in satellite images [25] and ranges between −1 to 1, with values below zero indicating non-water bodies and near-zero values indicating shallow water. Mathematically, NDWI is notated as follows, and computed with the function of “ee.Image.normalizedDifference” (Fig. 8).

$$NDWI=\frac{\rho_{NIR}-\rho_{r}}{\rho_{NIR}+\rho_{r}}$$

#### Further steps

Performing both the image pre-processing and feature extraction steps detailed in this method paper will produce processed satellite images that will enable machine learning for the classification of coastal ecosystems. The specific steps to perform machine learning for this purpose are beyond the scope of this method paper. However, other studies exist that have described the various algorithms that can be used for coastal ecosystem mapping based on machine learning in GEE [[26], [27], [28]]. The methodological steps detailed in this study can also be adapted for purposes beyond ecosystem classification based on machine learning, for instance, for bathymetry [29], and seawater quality and coastal biodiversity monitoring [30,31]. The general methodological steps provided in this paper were designed to perform satellite image pre-processing and feature extraction directly in GEE that can be readily applied for further spatial analysis as required by practitioners, such as area managers, for various environmental management needs. The development of the readily adaptable workflow is essential to empower and enable local area managers to conduct monitoring of their area, particularly due to increasing stressors caused by anthropogenic activities and environmental changes that are impacting these ecosystems and the services they provide [[32], [33], [34]].

### Method validation

To assess the practicality of this method, validation was performed by inviting practitioners to evaluate the steps described above. Invitations to participate in this validation step were sent to practitioners within the network of the authors of this paper, who were contacted individually to explain the purpose of this validation step. Out of five invitations sent, three practitioners agreed to participate and gave their written consents prior to the methods trial.

During the validation step, the practitioners were asked to follow the steps described above in the Method Details section, after which their feedback was collected. As part of the feedback, practitioners were asked to provide a Likert scale rating of 1 to 5 on the user-friendliness of the described methodology, with 5 being very easy to use. The participating practitioners were also asked to provide comments on the utility of the workflow. All of these are displayed in Table 1. Although the validation phase involved a limited number of participants, the validation results confirmed the accuracy and reliability of the proposed workflow. Nevertheless, we recognize that diverse practitioners may necessitate modifications to the pseudocodes presented here to accommodate their specific application requirements. Consequently, we have made the image preprocessing and feature extraction codes publicly available as an open-source resource [35], facilitating customization and adaptation for specialized research and operational needs.

**Table 1:** Participating practitioners’ feedback.

**Participant**	**Feedback**
#1 (Practitioner from local government body).	• Rating of user-friendliness: 4. • Experience in using GEE: 1 year. • Comment: “*The methodology is clear enough.*”.
#2 (Practitioner from non-governmental organization).	• Rating of user-friendliness: 3. • Experience in using GEE: None. • Comment: “*As a first-time user of GEE, it takes some time for me to get used to this methodology, especially as I am not familiar with Python. However, overall, the steps are relatively easy to follow.*”.
#3 (Practitioner from local government body).	• Rating of user-friendliness: 5 • Experience in using GEE: 1 year, with relevant experience as GIS developer of 10 years. • Comment: “*Very easy to follow, and I did not encounter error.*”.

### Limitations

The methodological steps described here require basic understanding on the use of Python programming language, and thus may provide a barrier to its adoption by practitioners who are not familiar with Python as indicated by a comment provided by one of the participants during the validation process. However, open-access resources are available widely (e.g. [[11], [12], [13]]) to enable familiarization with the programme. In addition, the operation of GEE will require a working internet connection, hence limiting the applicability of our approach only to those having access to the internet.

### Ethics statements

For the validation step, invited practitioners provided their written informed consent prior to their participation, ensuring their anonymity. Personal or demographical data were not collected from the participating practitioners.

## Credit author statement

**Ahmad Badruzzaman**: Conceptualization, Methodology, Writing – Original Draft. **Prawesti Wulandari** and **Sainal Sainal**: Writing – Review and Editing. **Matthew Ashley**: Supervision, Writing – Review and Editing. **Susan Jobling** and **Melanie C. Austen**: Supervision, Writing – Review and Editing, Funding Acquisition. **Radisti A. Praptiwi**: Conceptualization, Project Administration, Supervision, Writing – Original Draft, Writing – Review and Editing, Funding Acquisition.

## Declaration of interests

The authors declare that they have no known competing financial interests or personal relationships that could have appeared to influence the work reported in this paper.

## Data Availability

No data was used for the research described in the article.
